# Cultural competence in United States medical education: a scoping review of implementation and evaluation practices

**DOI:** 10.5116/ijme.69c3.e171

**Published:** 2026-04-09

**Authors:** Neeti Swami, Haley Lewsey, Angelica Nibo, Radha Patel, Stephanie Stroever, Lauren Cobbs

**Affiliations:** 1 Department of Medical Education, Texas Tech University Health Sciences Center School of Medicine, USA

**Keywords:** Cultural competence curricula, best evidence medical education, medical education research, instructional design, evalua-tion

## Abstract

**Objectives:**

The objective is to identify what is needed in Cultural Competence Curricula in medical schools and suggest a framework for evidence-based curricula that can be flexibly applied.

**Methods:**

We conducted a scoping literature review of Cultural Competence Curricula in United States medical schools. After reviewing 160 articles, 77 met inclusion criteria for analysis. We collected qualitative data on curricula described in each article to analyze elements of curriculum structure, evaluation, and study design.

**Results:**

Our results illustrate a high prevalence of structure styles conducive for quality learning, including longitudinality, integration, incorporation into clinical training, and experiential learning. The most common method for evaluating student learning was student self-evaluation with few programs performing reevaluations or utilizing patients as evaluators. Of knowledge, attitudes and skills, skills were least evaluated. Curricula with higher self-reported efficacy used a greater proportion of self-evaluations, while ones with lower self-reported efficacy used more external evaluations. Quasi-experimental study designs were more common in curricula with high self-reported efficacy.

**Conclusions:**

Curriculum developers across the world can improve implementation of Cultural Competency Curricula by maximizing the quantity of structural components, having higher quality of evaluation, and connecting with the local community surrounding their medical school. To develop a robust curriculum, we encourage longitudinal multi-component learning in integrated courses evaluated via experimental and quasi-experimental study designs.

## Introduction

Cultural Competence is a concept of relevance to medical schools all across the world as the impact of globalization continues to increase. Being a country with a diverse population, cultural competence has gained importance in the United States (U.S.) and its medical schools throughout time. Cultural competence is taught using many different structural formats across the U.S. that can be used in many different environments.

### A Brief History of Cultural Competence

The notion of cultural competence was originally rooted in the social movements of the 1960s and 1970s. This tumultuous time brought attention to racial and cultural disparities through the civil rights movement, the gay rights movement, women’s liberation, environmentalism, and counterculture. The need to operate within a multicultural society led to the term cultural competence, coined in 1989 by Terry L. Cross.^1^ Following desegregation, the reality of unresolved injustices and health inequity in the United States was evidenced by the 2002 Institute of Medicine publication Unequal Treatment: Confronting Racial and Ethnic Disparities in Health Care which caused a shift in cultural group-specific knowledge to patient-centered frameworks.^2^ Subsequently, in 2003, the Liaison Committee on Medical Education (LCME) required medical students to demonstrate cultural competence upon graduation to ensure that future physicians are prepared to provide quality care to historically underserved groups, sparking nationwide reform in cross-cultural medical education by establishing a formal curricular standard.^3^^,^^4^

Cultural Competence Curriculum (CCC) is commonly defined in a medical context as education regarding knowledge, skills, and attitudes conducive for cross-cultural communication.^5^ This definition has provided a conceptual basis for the implementation of programs aimed at establishing cultural competence in their students. To acknowledge the diversity present throughout various populations, the definition of culture in education has expanded and includes race/ethnicity, gender, sexuality, religion, disability, and the historical and socioeconomic context that have led to health inequity for underserved groups.^6^^-^^8^

Since undergraduate medical education exists within varying political climates, the value and quality of cultural competence education is determined by the discretion of individual programs.^4^ For example, the approach to cultural diversity varies by program, and there is controversy around ‘political correctness’ and implementation of social justice curricula.^9^^-^^11^ Additionally, “competence” implies a stagnant point in knowledge acquisition, so many programs prefer the term “cultural humility” to embrace lifelong learning because culture is ever-changing.^12^^-^^14^

### Observations in Current Literature

The literature reveals a vast array of definitions of culture and regional needs pertaining to the local communities surrounding the schools.^6^^-^^8^ This also means there is huge variability in the format of how CCC is taught. This increases challenges for standardization of development, implementation, and assessment of CCC, causing difficulty in identifying best practices and determining the impact of such practices on patient outcomes and health disparities.^15^ This also leads to many different educational structures. The literature currently has limited data that holistically shows and compares these curriculum structures while also connecting them with student outcomes and a best model for CCC implementation.

Medical CCC structure varies in terms of course length, frequency, rigor, level of integration, level of interaction, and experiential learning.^16^^-^^18^ However, there has been a historical evolution from the typical one-day didactic interventions of the 2000s and early 2010s toward instruction spread across up to four years.^16^^,^^19^^-^^21^ These longitudinal curricula also facilitate active learning by incorporating interactive learning activities due to increases in supporting evidence.^16^^,^^19^^-^^21^ Some cultural competence curricula remain isolated from basic science and clinical skills curricula while others have adopted an embedded approach.^17^^,^^22^ Modern curricula are increasingly emphasizing community partnership and cultural immersion with some regularly invite community members to speak on their experiences^14^ and others having students walk in the shoes of their fellow community members.^18^ Overall, these changes represent a trend toward curricular recognition of the complexity and intersectionality needed to guide student understanding of the sociocultural dynamics that impact patients.^17^^,^^23^^,^^24^

Success is often determined by formal or informal evaluation. Medical schools evaluate their CCC by utilizing either their students’ self-perceptions of their abilities, external evaluations assessed by another individual, team, third-party, and/or accreditation body.^16^^,^^25^^,^^26^ Several evaluation formats exist including surveys, focus groups, interviews, quizzes, tests, Objective Structured Clinical Exams (OSCE), thematic analyses, patient assessments, and community projects.^16^^,^^25^^,^^26^ While most articles include post-implementation performance or student-made reports of improvement, comprehensive evaluation methods and assessment of application of material in real-life scenarios is lacking.

In fact, current literature includes few instances of reevaluation or patient evaluation of students’ learning of cultural competence.^27^ Evaluations are instead often written and assessed by medical personnel such as faculty, medical students, and trained standardized patient actors, but not patients. Little to no programs utilize evaluations from patients due to challenges in asking patients to evaluate students which include obtaining Institutional Review Board (IRB) approval, risks for coercion, and limited time, resources, and patient interest. Hospital and clinical medicine runs off productivity which can discourage using extra time to acquire feedback from patients regarding learner performance, and delivering feedback upstream is often not a straightforward process for medical staff.^28^^,^^29^ Many patients opt out because they believe their feedback will not be used, rendering the process meaningless for making change.^29^ However, a few programs found promising alternatives by inviting people representing specific cultural backgrounds to act as patients and evaluate medical students.^6^^,^^30^^,^^31^ Without a means of evaluating retention of material over time or patient response to students who have participated in CCC, we may not be ensuring that future physicians will practice cultural competence that patients feel truly benefits them. Studies suggest medical students may overestimate their culturally sensitive skills compared to knowledge assessments and that all CCC has had little to no effect on patient satisfaction or outcomes.^15^^,^^32^ This indicates that despite curricular developments over the past few decades, current non-patient-centered assessments remain unsuccessful at pointing out the cross-cultural gaps that patients identify as needing improvement, perpetuating physician unpreparedness to respond to real-world needs.

### Review Question and Objectives: Identifying Components of Effective CCC

Upon starting medical school, we embarked on our professional journeys with a desire to become physicians capable of creating a more equitable healthcare system.^33^^,^^34^ As a result, we took note of how cross-cultural instruction and social determinants of health were embedded into the medical school curriculum. We found that successful CCC are made up of many elements that each contain variability, and saw a gap in the literature where best practices for curriculum structure and evaluation need further identification.^17^^,^^21^^,^^35^^,^^36^ Thus, our review focuses specifically on structural components and their relationship to the efficacy of CCC.

We examined the manner in which educators design CCC for student benefit in the short-term and patient benefit in the long-term. Since the variability in training and evaluation methods makes it difficult to identify best practices and the effects on patient outcomes, this paper focuses on the following research question: What are the current needs and ways to improve the structural implementation of evidence-based CCC at United States medical schools? Related objectives include: (1) to elucidate the current state of CCC educational formats in U.S. medical schools, (2) to identify the current key structural components of CCC, given the attempted changes over time in CCC delivery, (3) to determine how to appraise the efficacy of key structure components and (4) to observe whether quality of assessment can be attributed to study design.

Existing scoping reviews of the structure of CCC describe evaluation methods and the prevalence of curricular components in narrative synthesis without cross-comparison between elements.^27^^,^^37^ A 2023 review from Li and colleagues synthesized cultural competence frameworks and models and created an actionable guide using an Acceptance Commitment and Training (ACT) cultural model.^23^ Our scoping review similarly investigates structural elements of CCC, but is innovative in its analysis of the relationship between those elements and student learning outcomes. We suggest a framework that promotes teaching models with positive reports of efficacy in implemented CCC for other curriculum developers to reference when creating their own CCC. Though our framework has specific recommendations for the structure of CCC, there are successful curricula that do not fall strictly within the guidelines in our review.  In this review, efficacy is defined by the extent to which a program met their desired outcomes according to each included article. In addition, our review is comparative between papers and focused on efficacy in relation to unique elements such as curriculum structure and evaluation methods. It provides a robust qualitative analysis via descriptive statistics and offers suggestions for increasing patient evaluations. Thus, this paper focuses on curriculum structure, efficacy, and evaluation techniques of CCC; it does not delve into the content of the curricula at this time.

## Methods

### Search Strategy

**Figure 1:**
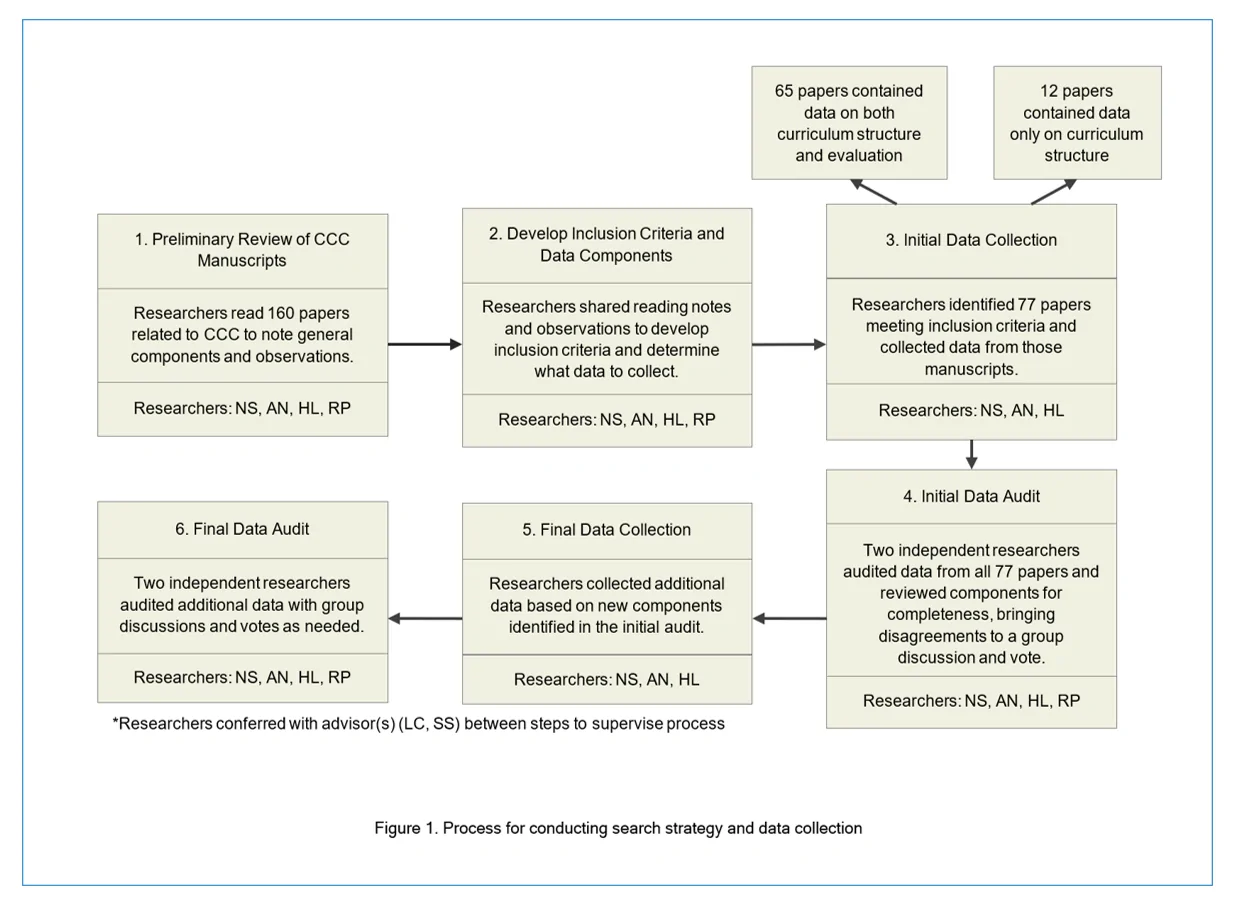
Process for conducting search strategy and data collection

We performed an initial search of Google Scholar, Scopus, Ovid, and PubMed aimed at locating published studies on the topic, with the latest review in March 2023 (see Figure 1 for search strategy and data collection process). The text words contained in the titles and abstracts of relevant articles, and the index terms describing the articles were used as key terms in the search strategy. We developed key words by finding index articles and identifying their key words, then refining the search iteratively to land on the best search strategy that would yield the most comprehensive results. Consistent keywords including “cultural competence curriculum,” “medical school,” “education,” and “cultural competence” were used in all databases along with various boolean operators. Other keywords included, but weren’t limited to, "cultural humility curriculum," “multicultural curriculum,” "curriculum," "training," "program," and “cultural education.” We also used citation analysis to further enhance our sample and continued reviewing until saturation was met. We also had routine meetings as the search was run to maintain consistency. During preliminary review of CCC articles, publication year, regional differences, public funding, course structure, content preferences, definitions of cultural competence, variance in teaching methodology, and reported outcomes were noted. Due to no human participants being directly involved in the methodology of this study, risks were considered minimal and no IRB approval was needed.

### Study Selection

After preliminary reading and discussion of 162 articles, we reached a consensus on elements and their operational definitions for qualitative data collection. Citations of these articles were collated and uploaded into EndNote 21 (Clarivate, London, UK) and duplicates removed. One-hundred sixty unique articles were identified in total. Inclusion criteria and data components were developed at this time. Per Joanna Briggs Institute (JBI) methodology, following initial data collection, articles were screened by two or more of our team members for assessment against the inclusion criteria for the review.38 Seventy-seven articles were included and eighty-three articles from the total 160 were excluded.

### Inclusion and Exclusion Criteria

All articles analyzed in the scoping review had a description of a CCC enacted in an individual U.S. Doctor of Medicine (M.D.) medical school. Only U.S. M.D. schools were included since all of their cultural competence curricula operate under LCME requirements. The purpose of this study was to identify the best approaches to teaching cultural competence topics in undergraduate medical education. Thus, the articles needed to have commented on either curriculum structure and/or evaluation on the efficacy of their program to be included in the study. Only studies published in English were included because the United States M.D. schools publish their articles primarily in English. Additionally, there were limited resources for translating and reviewing articles in other languages. Studies published since 1989 were reviewed as this was when the term cultural competence was coined, with the earliest included study being published in 1994.^1^ Articles that covered programs taking place across multiple medical schools, were non-M.D., or took place outside of medical school were excluded. Programs that did not give information on their curriculum structure or program evaluation were also excluded.

This scoping review included articles with both experimental and quasi-experimental study designs consisting of randomized controlled trials, non-randomized controlled trials, before and after studies, and interrupted time-series studies. In addition, analytical observational studies such as prospective and retrospective cohort studies and case-control studies were included. This review also included descriptive observational study designs like case series. Qualitative studies focused on qualitative data including, but not limited to designs such as phenomenology, grounded theory, qualitative description, and action research were not excluded.

Seventy-seven articles covering CCC structure and/or evaluation of outcomes were identified. Of these 77 articles, 65 articles contained information on both curriculum structure and evaluation.

### Data Extraction and Synthesis

For initial data collection, two or more of our team members independently extracted qualitative data from the articles included in the scoping review by using a review methodology based on the JBI scoping methodology.^38^ To maintain consistency between team members during data extraction and synthesis, all team members were trained to collect data based on agreed upon operational definitions based on each term in the context of CCC, expanded upon in Table 1. Using a qualitative approach to document these previously defined elements, final extracted curriculum data included details on structural components, attendance requirements, local community involvement, study design, methods of evaluation, evaluated constructs of cultural competence, and efficacy remarks. “Efficacy” in this review is defined by the extent to which programs achieved their desired outcomes. For the purpose of analysis, curriculum structure components included Integration, Longitudinality, Clinical Training, Experiential Learning, Interactive Activity, Passive Learning, Mandatory, and/or Optional. Study design elements included Pre-Curriculum Evaluation, Quasi-Experimental and Post-Curriculum Evaluation. Methods of evaluation included the elements of External Evaluation, Self-Evaluation, Reevaluation, and Patient Evaluation. The elements of evaluated cultural competence constructs included Knowledge, Skills, and Attitudes. These findings were tabulated on a master spreadsheet. All data underwent a final audit by at least two trained researchers. Any disagreements between independent reviewers were arbitrated by a third reviewer. The spreadsheet data was then reviewed with descriptive statistics and depicted graphically to summarize and compare the results.

## Results

The presentation of results includes a descriptive summary of data collected on curriculum structure and program evaluation, followed by summaries of the cross-comparisons performed between elements. For the purpose of this review, “structural components” refer to different aspects of a curriculum’s design, not limited to teaching methods and course duration. For analysis, “efficacy” was defined by the curriculum’s low, medium, or high level of achievement of the article authors’ desired outcomes.

### Descriptive Summary of Curriculum Structure

Data on curriculum structure was divided into nine elements: Mandatory and Optional attendance, involvement of a Local Community near the school, and a focus on Integration, Longitudinality, Clinical Training, Experiential Learning, Interactive Activity, and Passive Learning (see Table 1). Throughout this paper, these elements are referred to as “structural components.” Analysis of curriculum structure demonstrated Passive Learning to be most commonly utilized, with presence in 97% (n=77) of curricula. Longitudinality, Clinical Training, and Integration with other existing courses were the least commonly utilized, each found in less than 55% (n=77) of curricula. All schools used at least two of the six main components, and 19% (n=77) of schools used all six. The most common quantity of structure components incorporated was four components, present in 31% (n=77) of curricula.

### Descriptive Summary of Curriculum Evaluation

Analysis of curriculum evaluation was divided into three categories: Method of Evaluation, Constructs Evaluated, and Efficacy.

Data on competence assessment was gathered by observation of the cultural competence constructs that programs evaluated. The category ‘Constructs Evaluated’ was further divided into evaluation of Knowledge, Skills, and Attitudes. Of these constructs, Attitudes were the most evaluated at 94% (n=77), while Skills were the least evaluated at 72%.

Method of Evaluation was divided into Study Design and four assessments of student learning: External Assessment, Self-evaluation, Reevaluation, and Patient Evaluation. Self-evaluation was found to be the most common assessment method, employed in 83% (n=65) of curricula, while External Evaluation was used in 68% of curricula. Reevaluation and Patient Evaluation were utilized the least, at 15% (n=65) and 6.2%, respectively.

Regarding study design, the majority of programs utilized either a Post-Curriculum Evaluation only or a Quasi-Experimental (pre and post) design, with 52% (n=65) and 45% usage respectively. Very few programs used a Pre-Curriculum Evaluation only or a Randomized Controlled Trial study design, at less than 1.5% (n=65) usage each.

### Cross-Comparison: Structure and Efficacy Findings

Cross-comparison of curriculum structure to efficacy revealed the most commonly implemented combinations of structure components in relation to the level of efficacy.

**Table 1:** Literature review summary of cultural competence articles published between 1994 and 2023 categorized by article

Authors	Year	Medical School	Structural Components*	Attendance Requirement	Local Community Involvement	StudyDesign*	MethodofEvolution*	Constructs of Cultural Competence Evaluated	Efficacy
Allen, et al.	2020	University ofLouisville School of Medicine	Integration, Interactive Activity, Passive Learning	Mandatory	Kentucky smokers	Quasi	Self	K, A	3
Alli, et al.	2023	Mayo ClinicAlix School of Medicine	Experiential Learning,Interactive Activity,Passive Learning	Mandatory	None	Post	External, Self	K, A	3
Betancourt & Cervantes	2009	Harvard Medical School	Integration, Longitudinality, Clinical Training, Interactive Activity,Passive Learning	Mandatory	None	Post	External, Reeval	K, S, A	3
Bi, et al.	2020	University of Chicago Pritzker School of Medicine	Experiential Learning,Interactive Activity,Passive Learning	Mandatory	LGBT andracial/ethnic minorities	Quasi	Self	K, S, A	3
Blue, et al.	2005	Medical University of South Carolina	Integration, Clinical Training, Experiential Learning, Passive Learning	Mandatory	None	Post	External, Self	K, A	1
Borowsky,et al.	2021	University of California San Francisco School of Medicine	Interactive Activity,Passive Learning	Mandatory	None	Quasi	External, Self, Reeval	K, A	3
Brainin-Rodriquez	2001	University of Hawaii John A. Burns School of Medicine	Integration, Clinical Training, Experiential Learning, InteractiveActivity, Passive Learning	Mandatory	None	Post	Self	K, S, A	3
Bright & Nokes	2019	Tufts University School of Medicine	Interactive Activity,Passive Learning	Optional	None	Quasi	Self	K, S, A	3
Burson, et al.	2022	Perelman School of Medicine, University of Pennsylvania	Interactive Activity,Passive Learning	Mandatory	None	Post	External, Self, Reeval	K, A	2
Carpenter,et al.	2011	University of Hawai‘i John A. Burns School of Medicine	Integration,Longitudinality, Clinical Training, Experiential Learning, InteractiveActivity, Passive Learning	Mandatoryand OptionalElements	NativeHawaiians	Quasi	External, Self	K, S, A	3
Carter, et al.	2006	Uniformed Services University of the Health Sciences	Integration, Clinical Training, InteractiveActivity, Passive Learning	Mandatory	None	Quasi	Self	K, A	3
Coria, et al.	2013	Geisel School of Medicine	Integration,Longitudinality, Clinical Training, Experiential Learning, InteractiveActivity, Passive Learning	Mandatory	None	Post	External	K, S, A	3
Crandall, et al.	2003	Wake Forest University School of Medicine	Integration,Longitudinality,Experiential Learning,Interactive Activity,Passive Learning	Optional	None	Quasi	Self	K, S, A	3
Crocker, et al.	2019	University of Alabama School of MedicineBirmingham	Interactive Activity,Passive Learning	Mandatoryand OptionalElements	None	Post	External, Self	K, S, A	2
Crosson, et al.	2004	New Jersey Medical School	Integration,Longitudinality,Experiential Learning,Interactive Activity,Passive Learning	Mandatory	None	Quasi	Self, Reeval	A	3
DallaPiazza, et al.	2020	Rutgers New Jersey Medical School	Integration,Longitudinality, Clinical Training, Experiential Learning, InteractiveActivity, Passive Learning	Mandatory	None	Post	External, Self	K, S, A	3
Dao, et al.	2017	Perelman School of Medicine at the University of Pennsylvania	Experiential Learning,Interactive Activity,Passive Learning	Mandatory	None	Post	Self	K, S, A	3
Denizard- Thompson, et al.	2021	Wake Forest School of Medicine	Integration, Clinical Training, InteractiveActivity, Passive Learning	Mandatory	None	Quasi	Self, Reeval	K, S, A	3
Dhanani, et al.	2022	Stanford Schoolof Medicine	Interactive Activity,Passive Learning	Optional	None	Quasi	Self	K, S, A	3
Dogra &Karnik	2003	University of Illinois Colleges of Medicine atChicago and Urbana- Champaign	Interactive Activity,Passive Learning	Optional	None	C/S Pre	External, Self	K, A	1
Ellison, et al.	2021	LSU Health New Orleans School of Medicine	Longitudinality, Interactive Activity, Passive Learning	Mandatory	New Orleans’ segregation and racism	Quasi	Self	K, A	3
Fracica, et al.	2016	Mayo Medical School Minnesota	Experiential Learning,Interactive Activity,Passive Learning	Optional	Somalipopulation in Minnesota	Quasi	External, Self	K, A	2
Genao, et al.	2011	Emory University School of Medicine	Integration, Clinical Training, InteractiveActivity, Passive Learning	Mandatory	None	RCT	External	K	3
Godkin & Weinreb	2001	University of Massachusetts Medical School	Integration,Longitudinality, Clinical Training, Experiential Learning, InteractiveActivity, Passive Learning	Optional	Immigrant and refugee groups	Post	Self	K, S, A	3
Godley, et al.	2020	University of North Carolina School of Medicine	Experiential Learning,Interactive Activity,Passive Learning	Optional	Black southerners	Post	External, Self	K, A	3
Gonzalez,et al.	2021	Albert Einstein College of Medicine	Interactive Activity,Passive Learning	Mandatory	None	Post	External, Self	K, S, A	3
Green, et al.	2017	Harvard Medical School	Integration,Longitudinality, Clinical Training, PassiveLearning	Mandatory	None	Quasi	Self	K, S	2
Green, et al.	2007	Harvard Medical School	Integration,Longitudinality,Experiential Learning,Interactive Activity,Passive Learning	Mandatory	None	Post	External,Patient	K, S, A	3
Greene & Scott	2021	Morehouse School of Medicine	Integration, Experiential Learning, PassiveLearning	Mandatory	Deaf/hard of hearing	Quasi	External	K, S, A	3
Griswold, et al.	2007	The State University of New York	Experiential Learning,Interactive Activity,Passive Learning	Optional	International refugees	Post	External, Self	K, S, A	3
Hearn & Hearn	2020	University of Michigan Medical School	Interactive Activity,Passive Learning	Mandatory	None	Quasi	External, Self	K, S, A	3
Hutchins,et al.	2014	University ofWisconsin– Madison’s Global Health Institute	Integration, Experiential Learning, InteractiveActivity, Passive Learning	Optional	None	Post	Self, Reeval	K, S, A	3
Kenison,et al.	2017	Harvard Medical School	Integration,Longitudinality, Clinical Training, Experiential Learning	Optional	None	Post	External	K, S, A	1
Khoury, et al.	2022	SUNY Upstate Medical University	Integration, Clinical Training, InteractiveActivity, Passive Learning	Mandatory	None	Quasi	External	S, A	3
Kitzes, et al.	2007	University of New Mexico School of Medicine	Integration,Longitudinality, Clinical Training, Experiential Learning, InteractiveActivity, Passive Learning	Mandatory	NativeAmerican/rural/Hispanicpopulation	Post	External, Self	K, S	3
Lie, et al.	2006	University of California, Irvine, School of Medicine	Integration, Longitudinality, Clinical Training, Experiential Learning, InteractiveActivity, Passive Learning	Mandatory	None	Post	Self	K, S, A	3
Lie, et al.	2010	University of California,Irvine School of Medicine	Integration, Clinical Training, Experiential Learning, InteractiveActivity	Mandatory	Latino	Post	External, Self	K, S, A	3
Litzelman & Cottingham	2007	IndianaUniversity School of Medicine	Integration,Longitudinality, Clinical Training, Experiential Learning, InteractiveActivity, Passive Learning	Mandatory	None	Post	External, Self, Patient	S, A	3
Lombardero, et al.	2022	University of Nevada, Reno, School of Medicine	Longitudinality, Clinical Training, InteractiveActivity, Passive Learning	Optional	None	Quasi	External, Self	K, S, A	3
Loue, et al.	2015	Case Western Reserve University School of Medicine	Longitudinality,Experiential Learning,Interactive Activity,Passive Learning	Mandatory	None	Post	Self	K, S, A	1
Lubimir& Wen	2011	University of Hawai‘i John A. Burns School of Medicine	Clinical Training,Interactive Activity,Passive Learning	Mandatory	None	Post	External, Self	K, S, A	3
McElfish,et al.	2017	University of Arkansas for Medical Sciences Northwest	Clinical Training,Experiential Learning,Interactive Activity,Passive Learning	Optional	Marshallesemigrants	Quasi	External, Self	K, S, A	3
Miller & Green	2009	Harvard Medical School	Integration,Longitudinality,Interactive Activity,Passive Learning	Mandatory	Bilingual Latina women	Post	External, Self	K, S, A	3
Nora, et al.	1994	Rush Medical College	Longitudinality,Experiential Learning,Interactive Activity,Passive Learning	Optional	Hispanic Health Alliance	Quasi	External, Self	K, S, A	3
Novak, et al.	2022	University of Southern California Keck School of Medicine	Interactive Activity,Passive Learning	Mandatory	None	Post	External, Self	K, A	3
Offei-Dua,et al.	2022	Washington University in St. Louis School of Medicine	Experiential Learning,Interactive Activity,Passive Learning	Mandatoryand OptionalElements	Neighborhood tour, African American/ Latinx/ refugee health	Post	External, Self, Reeval	K, S, A	1
Omoruyi,et al.	2018	University of Texas Health Science Center at Houston	Integration, Clinical Training, Experiential Learning, InteractiveActivity, Passive Learning	Mandatory	None	Quasi	External, Self	K, S, A	3
Ona, et al.	2020	Tufts University School of Medicine	Interactive Activity,Passive Learning	Optional	None	Quasi	External, Self	K, S, A	3
Ortega, et al.	2021	University ofIllinois College of Medicine	Clinical Training,Experiential Learning,Interactive Activity,Passive Learning	Optional	None	Quasi	External, Self	K, S, A	3
Perlman& Stagnaro-Green	2010	New Jersey Medical School	Integration,Longitudinality, Clinical Training, Experiential Learning, InteractiveActivity, Passive Learning	Mandatory	Alternative medicinecommunity	Post	External	K, S, A	2
Railey, et al.	2022	Duke University School of Medicine	Clinical Training,Passive Learning	Mandatory	None	Quasi	Self, Reeval	K, S, A	1
Robins, et al.	1998	University of Michigan Medical School	Integration, Interactive Activity, Passive Learning	Mandatory	None	Post	External	K, S, A	3
Sarmiento,et al.	2016	University of Michigan Medical School	Longitudinality,Experiential Learning,Interactive Activity,Passive Learning	Mandatory	Local disability group	Post	Self	K, A	3
Shapiro, et al.	2006	University of California Irvine, School of Medicine	Longitudinality, Clinical Training, Experiential Learning, PassiveLearning	Optional	None	Post	External, Self	K, S, A	2
Shield, et al.	2011	Alpert Medical School of Brown University	Integration, Longitudinality, Clinical Training,Experiential Learning,Interactive Activity,Passive Learning	Optional	None	Quasi	External, Self	K, A	3
Smothers,et al.	2018	Duke University School ofMedicine	Integration, Clinical Training, InteractiveActivity, Passive Learning	Mandatory	None	Quasi	Self	A	3
Swanberg,et al.	2015	OaklandUniversityWilliamBeaumont School of Medicine	Integration,Longitudinality, Clinical Training, Experiential Learning, InteractiveActivity, Passive Learning	Mandatory	None	Post	Self	K, A	3
Symons, et al.	2009	State Universityof New York (SUNY) at Buffalo	Integration,Longitudinality, Clinical Training,Experiential Learning,Interactive Activity,Passive Learning	Mandatory	Local disability groups	Quasi	External, Self, Patient	K, S, A	3
Thew, et al.	2013	University of Rochester School of Medicine and Dentistry	Experiential Learning,Interactive Activity,Passive Learning	Optional	None	Post	Self, Reeval	K, S, A	3
Turner & Farquhar	2008	College of Human Medicine (CHM) at Michigan State University	Integration,Longitudinality, Clinical Training, Experiential Learning, InteractiveActivity, Passive Learning	Mandatory	None	Post	External, Self, Reeval	S, A	3
Wong & Omori	2016	University ofHawai‘i John A. Burns School of Medicine	Integration,Longitudinality, Clinical Training, Experiential Learning, InteractiveActivity, Passive Learning	Mandatory	HomelessHawaiian island populations	Post	External	K, S, A	3
Woodard,et al.	2012	University of South Florida Health, Morsani College ofMedicine	Integration, Clinical Training, Experiential Learning, InteractiveActivity, Passive Learning	Mandatory	Communitysite visits	Quasi	External, Self, Reeval, Patient	K, S, A	3
Worden & Ait- Daoud Tiouririne	2018	University ofVirginia School of Medicine	Integration, Experiential Learning, InteractiveActivity, Passive Learning	Mandatory	None	Post	External	A	1
Zanetti	2014	University of Massachusetts Medical School	Clinical Training,Experiential Learning,Interactive Activity,Passive Learning	Mandatory	None	Quasi	External, Self	K, S, A	3
Zanetti, et al.	2011	University of Massachusetts Medical School	Longitudinality, Clinical Training, Experiential Learning, PassiveLearning	Optional	Recentimmigrants	Quasi	External, Self	S, A	3
Albritton& Wagner	2002	Medical College of Georgia	Integration,Longitudinality,Experiential Learning, Passive Learning	Optional	Center forlocal migrantworkers	N/A	N/A	N/A	N/A
Ambrose,et al.	2014	University ofHawai‘i John A. Burns School of Medicine	Longitudinality, Clinical Training, Experiential Learning, InteractiveActivity, Passive Learning	Optional	None	N/A	N/A	N/A	N/A
Bussey-Jones, et al.	2005	Emory University School ofMedicine	Integration, Clinical Training, Experiential Learning, PassiveLearning	Mandatory	None	N/A	N/A	N/A	N/A
Eddey& Robey	2005	Matheny Medical and Educational Center	Longitudinality, Clinical Training, Experiential Learning, InteractiveActivity, Passive Learning	Mandatory	Local disability organizations	N/A	N/A	N/A	N/A
Gupta, et al.	2020	Mayo Clinic Alix School ofMedicine Arizona	Integration,Longitudinality, Clinical Training, Experiential Learning, InteractiveActivity, Passive Learning	Mandatory and OptionalElements	None	N/A	N/A	N/A	N/A
Kumagai,et al.	2009	University of Michigan Medical School	Integration,Longitudinality,Experiential Learning,Interactive Activity,Passive Learning	Mandatory	None	N/A	N/A	N/A	N/A
Lewis & Prunuske	2017	University of MinnesotaMedical School, Duluth	Experiential Learning, Passive Learning	Mandatory	Indigenous population	N/A	N/A	N/A	N/A
Lypson, et al.	2008	University of Michigan Medical School	Integration,Longitudinality,Interactive Activity,Passive Learning	Mandatory	None	N/A	N/A	N/A	N/A
Rogers, et al.	2016	Mayo Medical School	Experiential Learning,Interactive Activity,Passive Learning	Optional	Local disability groups	N/A	N/A	N/A	N/A
Schiff &Rieth	2012	University ofHawai‘i John A. Burns School of Medicine	Longitudinality, Clinical Training, Experiential Learning, InteractiveActivity, Passive Learning	Optional	None	N/A	N/A	N/A	N/A
Solotke, et al.	2017	Yale University School ofMedicine	Integration,Longitudinality,Clinical Training,Experiential Learning,Interactive Activity,Passive Learning	Mandatory	Local organizations	N/A	N/A	N/A	N/A
Thande, et al.	2019	Yale University School ofMedicine	Integration,Longitudinality,Experiential Learning,Interactive Activity,Passive Learning	Mandatory	LGBT patients	N/A	N/A	N/A	N/A

Key: Year= Year Published. K= Knowledge, S= Skills, A= Attitudes. C/S Pre= Cross-Sectional Pre-Curriculum Evaluation. Quasi= Quasi-Experimental. Post= Post-Curriculum Evaluation. RCT= Randomized Controlled Trial. 1= Low Efficacy. 2= Medium Efficacy. 3= High Efficacy. N/A= Not applicable due to no reported evaluation in paper.

*Operational Definitions: Integration= Program is integrated with existing curricula not already centering cultural competence (clinical skills, basic sciences, OSCEs, etc.). Longitudinality= Program lasts at least 1 school year; only counted by aspects of program mentioned in that article; frequency within total duration is not taken into account. Clinical Training= Program occurs during clinical training; optional preceptorships not included. Experiential Learning= Program has community involvement with projects and/or direct contact with members/institutions of the community; inside or outside the classroom. Interactive Activity= Program incorporates interactive activities inside the classroom (discussions, role-plays, case-studies, project-based learning, etc.). Passive Learning= Program incorporates passive learning methods (lectures, readings, videos, etc.).^69^ Quasi= quasi-experimental (pre- and post-implementation evaluation); Post= post-implementation evaluation only; C/S Pre= cross-sectional pre-implementation evaluation only; RCT= randomized controlled trial. External evaluation= not from the students’ own perspective (quiz, OSCE, peer evaluation, final project, qualitative analysis of a self-assessment, etc.); Self-Evaluation= students’ self perception of their abilities (survey, reflection assignment, focus group); Reevaluation= any second form of evaluation after any amount of time after conclusion of the program; Patient Evaluation= any consultation of patients or standardized patients coming from the target cultural group(s) in evaluation. Low Efficacy (1)= no/very limited achievement of the article authors’ desired outcomes; Medium Efficacy (2)= some/multiple article authors’ desired outcomes met; High Efficacy (3)= all of the article authors’ desired outcomes met.

Each of the 25 observed curriculum structure combinations were labeled A through Y from most to least frequently implemented. Of the five most frequent structure combinations, combination A (n=15: Integration, Longitudinality, Clinical Training, Experiential Learning, Interactive Activity, and Passive Learning) and combination E (n=5: Integration, Clinical Training, Interactive Activity, and Passive Learning) had the most evaluations with high efficacy (A with 80% and E with 100%). Meanwhile, combination B (n=10: Interactive Activity and Passive Learning) and combination C (n=9: Experiential Learning, Interactive Activity, and Passive Learning) had the most evaluations with low efficacy (B with 10% and C with 11%). Programs using five or six structural components did not have low efficacy reports.

Of the schools with documented Efficacy, 35% (n=65) had involvement with local cultural groups. The programs with a local aspect of content had a higher percentage of high efficacy evaluations than those without this aspect, at 87% (n=23) compared to 76% (n=42). Additionally, local-focusing programs had fewer low efficacy evaluations, at 4.4% (n=23) compared to 14% (n=42).

### Cross-Comparison: Evaluation and Efficacy Findings

The curricula evaluated themselves through only Self-evaluation, only External Evaluation, or through a combination of both Self-evaluation and External Evaluation. After cross-comparing curriculum evaluation and efficacy, analysis demonstrated High Efficacy Curricula to have the highest proportion of solely Self-Evaluations, at 35% (n=52). Medium Efficacy Curricula used a combination of Self and External Evaluation most often, at 67% (n=6), while using only Self-Evaluation the least, at 17%.

As for study design, only 1 High Efficacy curriculum (1.9%, n=52) used a Randomized Controlled Trial, and only 1 Low Efficacy curriculum (14%, n=7) used a Cross-Sectional Pre-Curriculum study. Post-Curriculum Evaluations were most common for Low and Medium Efficacy curricula, at 71% (n=7) and 67% (n=6), respectively. Quasi-Experimental study designs were most common for High Efficacy programs at 50% (n=52).

## Discussion

Countries around the world are comprised of countless communities, each with their own culture. Especially with increased globalization and diversification of communities, teaching medical providers how to understand and acknowledge the impact of their shared lived experiences is necessary for optimizing health outcomes. This review offers a robust analysis of the successes and limitations of Cultural Competence Curricula (CCC) in order to depict its current state and identify quality implementation frameworks for future use. These observations for strengths, weaknesses, and best practice can be applied to CCC curriculum around the world. Though most programs we observed incorporate passive learning and promote student self-reflection, our results suggest that many may have missed opportunities to implement more engaging learning formats, ensure long-term retention, and gather external input from affected populations.

### Structure: What’s Needed?

Over 30% of programs did not incorporate Integration, Longitudinality, and presence in Clinical Training into their CCC, suggesting that there is a widespread lack of these elements in CCC. The paucity of these structural components indicates that many programs lack opportunities for students to reinforce learning through repetition and usage in clinical rotations. Though not considered in this review, longitudinal CCC extending into graduate medical education is an additional opportunity for reinforcement.^39^^,^^40^ Furthermore, without integration and engagement with this content during clinical training, students may not be connecting cultural competence to their growing scientific and clinical knowledge or to real patients. CCC can implement discussion-based settings, cross-cultural content in science blocks, and activities in the clinical years to help students increase cognitive processing, repetition, and application of material.^41^

### Evaluation: What’s Needed?

Our findings suggest that programs do not consistently assess all three domains of cultural competence: knowledge, skills, and attitudes. Skills are assessed the least, which demonstrates that some programs may not be assessing or emphasizing the importance of cross-cultural communication strategies. This could also be due to difficulty acquiring enough diverse standardized patients and resources like interpreters for relevant OSCEs. Schools may have difficulty hiring people willing to work sporadically as independent contractors or difficulty budgeting for the high costs of their services.^42^^,^^43^ However, skills are not to be prioritized over knowledge and attitudes. Students should leave medical school with increased knowledge of cultural norms and self-awareness, having potentially challenged their long-standing views.^44^ Additionally, developing a wider knowledge base, such as a greater understanding of historical factors, will prepare students to face health disparities visible during their clinical encounters.^44^ Discordance between knowledge, skills, and attitudes could lead to reduced student preparedness and hinder patient-physician interactions.

Our results reveal that many programs heavily relied on self-evaluations by students which suggests that success is being measured by perceptions of students and/or faculty rather than affected patients. Though this allows students to be introspective, it could lead to faculty and students perceiving their competence as higher than the reality. The interpersonal nature of cultural competence may affect the validity of intrapersonal evaluations like self-evaluations.^45^ As demonstrated in our findings, the curricula that intentionally discuss challenges and were deemed low or medium efficacy utilized external evaluations more heavily. This may indicate that without outside input from affected populations or bodies of cultural knowledge, some areas for improvement may be missed. We encourage all programs to consider using external rather than self-evaluation methods in order to be self-critical.^45^ As mentioned by multiple papers, self-evaluations are subjective: while they reflect student perspective, it does not indicate measurable growth or excellence in applied skills. However, for programs who would like to use self-evaluation to encourage self-reflection, we also recommend adding external evaluations to gain valuable and measurable information alongside student insight.^46^^-^^48^

The purpose of these aforementioned measures is to improve patient outcomes. Our review depicts a lack of patient evaluations, which represents a need for more patient input as patient satisfaction is the ultimate determinant of efficacy. Though patient evaluation is lacking, it is challenging to correct without an established model that addresses the previously mentioned barriers to organizing a standardized patient pool representative of populations in CCC.^29^^,^^42^^,^^43^ However, some programs have successfully overcome this challenge. For example, a 2013 study by Thew et al. provides a successful example of patient recruitment as they recruited and compensated deaf volunteers from the local community to participate in role-play exercises with medical students.^49^ Using community volunteers reduces costs and risk of coercion, allows for schedules that are appropriate for the volunteers’ and learners’ time, and enables true representation of the target patient population(s) in the curriculum. Four of our included studies describe patient evaluation models that CCC developers believe created more comprehensive learning experiences and were all deemed successful overall.^6^^,^^31^^,^^50^^,^^51^ We encourage programs to reflect on the few existing examples of patient evaluation and create a model that is both generalizable and manageable for patients and educators.

### Cross-comparisons: Recommendations for Best Practice

**Figure 2:**
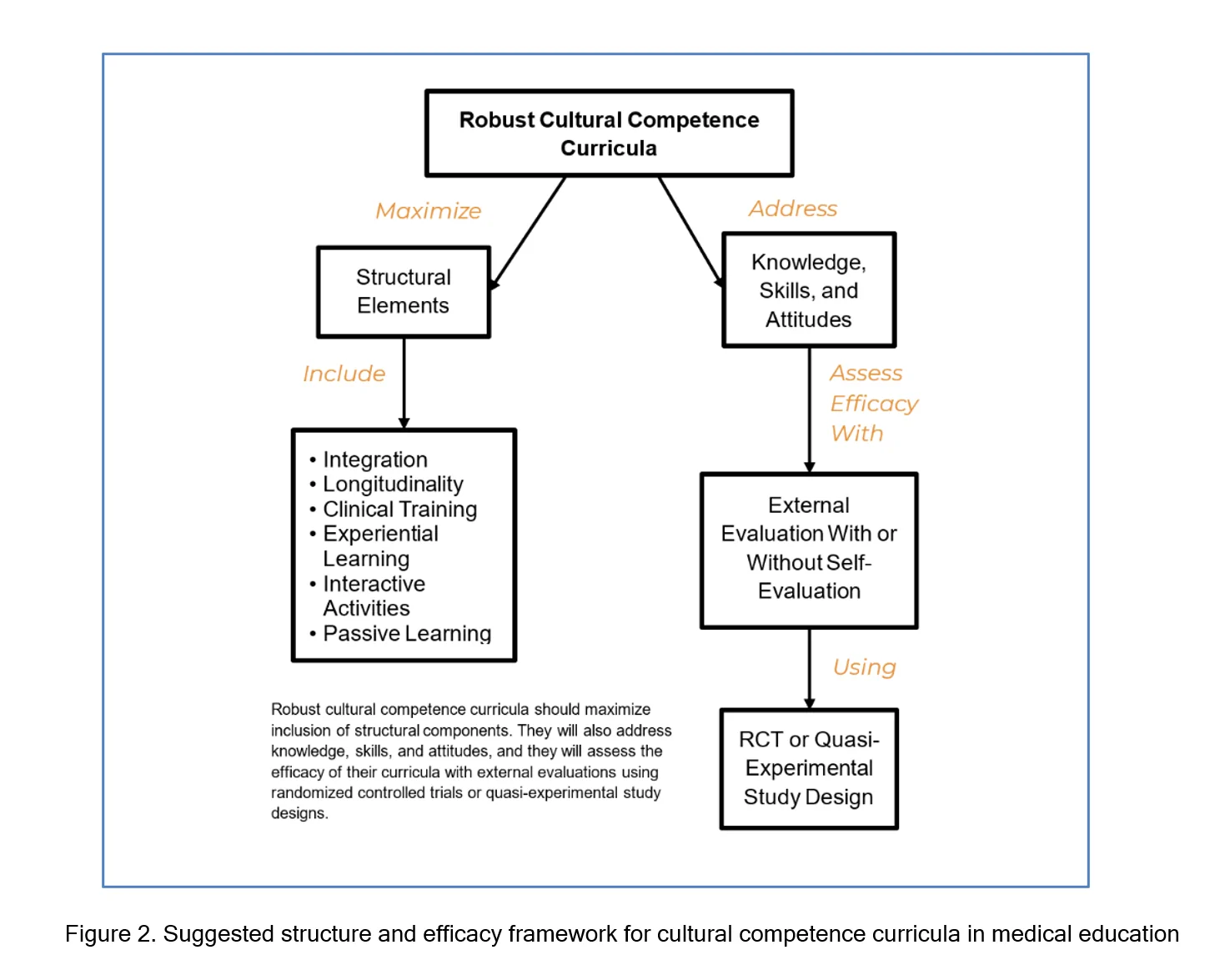
Suggested structure and efficacy framework for cultural competence curricula in medical education

Based on our findings, we have a suggested framework for curriculum structure and evaluation. Many of these findings are corroborated by other frameworks which share overlapping features such as the importance of community engagement, reviewing knowledge, skills, and attitudes, and different forms of student engagement.^17^^,^^23^^,^^52^^,^^53^ This framework, depicted in Figure 2, consists of maximal structure components, external evaluations jointly assessing knowledge, skills, and attitudes, and strong study design. It appears that successful programs maximize the quantity of structural elements, connect with the local community, and have higher quality evaluations.

Though our results suggest that several iterations of structure combinations can be effective, it seems that the quantity of components is a major factor. By incorporating more structural components, students will experience more varied reinforcement of cultural competence to provide them with more opportunities to engage with the material.^54^ Additionally, involvement of a community surrounding the school allows students to connect with real people, hear their stories, and see the real impact of cultural competence on them.^55^ Finally, we encourage programs to use higher quality study designs to build the strength of the literature for others to reference. Using pre- and post-evaluations or randomized controlled trials allows programs to establish a baseline, modify the curriculum according to the learning gaps specific to their students, and limit unaccounted variables affecting the success of a curriculum.

We acknowledge that there are other factors besides structure and efficacy that affect CCC improvement including faculty buy-in, student motivation or lack thereof, lack of diverse workforce, limited budget for interpreters, and access to cultural competence staff training.^56^^,^^57^ However, regarding structure and efficacy, this framework provides a more meaningful educational experience to students and may be significant for the continued betterment of cultural competence education in medicine.

### Global Importance and Relevance

Developing a medical school curriculum that teaches cross-cultural communication is important internationally. Every country has various communities with different lived experiences that can benefit from CCC. Certain populations are universally found around the world, such as those who are women, low socioeconomic status, disabled, rural, LGBTQ+, etc., who benefit from culturally competent care.^58^^-^^60^ Some elements of cultural competence such as social determinants of health, cross-cultural communication, and bias similarly affect patients worldwide.^61^^-^^63^

Moreover, like those in this review, programs across many countries often have specific populations with cultural considerations that impact health, such as migrant populations in Europe,^60^ aboriginal populations in Australia,^64^ and language barriers in South Africa.^65^ Based on the findings from our review, we recommend that curriculum developers involve these communities in their CCC so that students learn practical applications of cultural competence to their unique environment. Regardless of nation, teaching CCC is associated with better patient outcomes and can improve patient interactions,^66^^,^^67^ and many recognize the need to implement and improve CCC.^67^^,^^68^ Our paper provides curriculum structure recommendations on maximizing different learning methods, increasing the quality of student assessment, and encouraging better standards of study design that are applicable to designing and evaluating CCC anywhere in the world.

### Strengths and Limitations of This Review

As a scoping review, the authors acknowledge this work may not exhaustively include all scholarship on this topic. We do believe it is nonetheless a robust representation of the available literature in this area. Strengths of this review include the comprehensive methodology and depth of analysis specific to curriculum structure and program evaluation of the implementation of these curricula. Not only did we descriptively detail the usage of key structural components, but we connected and stratified usage by reported efficacy to highlight potential predictors of success for curriculum developers.

One of our main limitations was the qualitative, subjective nature of the articles, which prompted us to develop our own definitions of many elements. Some of these definitions, such as Low, Medium, and High Efficacy, were based on claims made by each article without objective standardization between articles for what constituted an efficacious curriculum. Observations were limited by what was explicitly stated, so it is possible that our data did not accurately represent a curriculum if its published paper lacked documentation of any aspect.

## Conclusions

Overall, curriculum development is extremely multifaceted. Our review suggests that the best practices include developing a longitudinal interactive curriculum that is integrated with clinical and basic sciences. It would also be beneficial for programs to work towards incorporating reevaluations and most importantly, patient evaluations. In the future, we plan to compose additional reviews that examine the content of the CCC articles and thematically analyze important aspects of cultural competence curricula that we have yet to discuss. In addition, we plan on introducing a patient assessment model to the Texas Tech University Health Sciences Center School of Medicine where patients from target cultural groups evaluate our students’ cultural competence. Future reviews can form other cross-comparisons, use our elements with newer publications, perform objective analyses of curriculum effectiveness, or examine the structural makeup and evaluation of CCC in more countries. As we continue improving upon cross-cultural education, we must remain steadfast in striving to empower patients to give us feedback and in looking for progress in quality of care.

### Acknowledgements

We would like to acknowledge and thank Texas Tech University Health Sciences Library for their support of our literature search.

### Conflict of Interest

The authors declare that there is no conflict of interest.
